# Thermal Adaptation Methods of Urban Plaza Users in Asia’s Hot-Humid Regions: A Taiwan Case Study

**DOI:** 10.3390/ijerph121013560

**Published:** 2015-10-27

**Authors:** Chen-Fa Wu, Yen-Fen Hsieh, Sheng-Jung Ou

**Affiliations:** 1 Department of Horticulture, National Chung Hsing University, Taichung 402, Taiwan; E-Mail: cfwu@dragon.nchu.edu.tw; 2 Department of Landscape and Urban Design, Chaoyang University of Technology, Taichung 413, Taiwan; E-Mail: sjou@cyut.edu.tw

**Keywords:** thermal adaptation, thermal comfort, coping, qualitative method

## Abstract

Thermal adaptation studies provide researchers great insight to help understand how people respond to thermal discomfort. This research aims to assess outdoor urban plaza conditions in hot and humid regions of Asia by conducting an evaluation of thermal adaptation. We also propose that questionnaire items are appropriate for determining thermal adaptation strategies adopted by urban plaza users. A literature review was conducted and first hand data collected by field observations and interviews used to collect information on thermal adaptation strategies. Item analysis—Exploratory Factor Analysis (EFA) and Confirmatory Factor Analysis (CFA)—were applied to refine the questionnaire items and determine the reliability of the questionnaire evaluation procedure. The reliability and validity of items and constructing process were also analyzed. Then, researchers facilitated an evaluation procedure for assessing the thermal adaptation strategies of urban plaza users in hot and humid regions of Asia and formulated a questionnaire survey that was distributed in Taichung’s Municipal Plaza in Taiwan. Results showed that most users responded with behavioral adaptation when experiencing thermal discomfort. However, if the thermal discomfort could not be alleviated, they then adopted psychological strategies. In conclusion, the evaluation procedure for assessing thermal adaptation strategies and the questionnaire developed in this study can be applied to future research on thermal adaptation strategies adopted by urban plaza users in hot and humid regions of Asia.

## 1. Introduction

In Taiwan, people spend about 90% of their daily time indoors [1]. Such long hours spent indoors limit contact with Nature and sunshine, and a long standing lack of physical and psychological relaxation can result in health deterioration effects such as myopia, obesity, depression and inability to concentrate [2,3,4,5,6,7,8,9,10,11,12]. In contrast, outdoor activities can significantly enhance physical and psychological health, including preventing myopia [2,3,4,5], reducing the likelihood of obesity [6,7,8], facilitating relaxation [8,9,10,11,12], and improving concentration [8]. Therefore, improving the quality of outdoor recreation can enhance the health of users. However, the gradual elevation of temperature has increased the discomfort of the outdoor environment. Between 1897 and 2008, the average temperature in Taiwan has increased by 0.8 ºC, and by 1.4 °C in the urban areas [13]. Compared to rural areas, the urban temperature is higher by an average of 6 °C [14], and its level of discomfort is correspondingly greater. Thus, improving thermal comfort in the urban outdoors is a critical issue for urban landscape planning.

Thermal adaptation surveys allow researchers to understand how people respond to thermal discomfort, and are crucial for improving thermal comfort in urban outdoor space. People respond differently to the various thermal environments that they are exposed to daily. When a thermal environment feels comfortable, people do not intentionally seek to change their behaviors, but when a thermal environment creates discomfort, they actively adopt thermal adaptation strategies to regain thermal comfort [15,16,17,18,19,20].

Studying on strategies of thermal adaptation also allows landscape designers to access people’s needs when facing thermal discomfort, and then create more suitable designs accordingly. Past studies have used different methods to measure thermal adaptation. Researches for indoor environments focus on observing clothing and analyzing the relationship between the changing clothing as a thermal adaptation strategy and temperature variation in indoor environments [21,22,23,24,25]. Some researchers used surveys to explore other thermal adaptation methods such as adjusting furniture, doors, windows, blinds, heaters, shades and fans in the building, or resting, drinking cold and hot beverages [20,26,27,28,29,30]. Studies on outdoor environments mainly investigate following approaches, which are adjusting psychological expectation, seeking roof covers, seeking tree shades, cooling under a pavilion, returning to indoor space, quickly moving out of the sun, removing an article of clothing, using an umbrella, wearing a hat, drinking cold and hot beverages, tying up the hair, and using a hand fan [19,31,32,33].

In most past thermal comfort studies, methods for determining thermal adaptation strategies involved questionnaires asking about the types of measures used in response to heat. Interview methods were both structured and non-structured. Non-structured methods mainly comprised open-ended items or observations about clothing changes to identify response strategies and latent constructs. Structured methods were used to evaluate thermal adaptation strategies and degree of agreement with the latent constructs, where respondents chose from a list of most frequently used behavioral adaptation strategies, such as seeking shades, adjusting clothing, and drinking beverages. However, few studies included questionnaire items on psychological adaptation [15,34]. Questionnaire items on thermal adaptation in hot and humid Asia often included adaptation variables such as seeking shade under a tree, removing an article of clothing, using an umbrella, wearing a hat, and drinking beverages, and were rather limited and therefore inadequate for fully determining the thermal adaptation strategies used by the respondents [16,19,23,31,32,33].

This study is an in-depth investigation to gain a comprehensive understanding of thermal adaptation strategies adopted by users confronted with outdoor thermal discomfort in hot and humid Asia, which is crucial for compensating for the gaps in the abovementioned studies. Previous thermal comfort studies and stress adjustment studies have found a wide variety of dimensions in behavioral and psychological adaptation strategies. Most thermal comfort studies focused on behavioral thermal adaptation strategies, and only a few studies focused on psychological adaptation strategies. Moreover, items related to psychological adaptation in questionnaire were somewhat scattered in wide variety of researches. Some of them focus on expectations [26], past experience [35,36], expectations and past experience [16], past experience and perceived control [32], product transfer and rationalization [34,37] and others [18].

Many previous thermal comfort studies required a survey of thermal adaptation strategies to determine people’s responses to thermal discomfort. However, compared to other regions, Asia has more hot and humid weather conditions. Therefore, the study of thermal adaptation strategies in urban outdoor space in hot and humid Asia is a crucial research topic [19,32,33]. Nevertheless, measurement items in previous studies were simplistic: Kao [34] and Wu [37] surveyed product transfer, rationalization, direct action, inside displace, outside displace and time displace by questionnaires in Alishan National Forest Recreation Area and Taichung Jingguo Green Pathway. Lin [32] surveyed seeking roof covers, seeking tree shade, removing an article of clothing, using an umbrella, wearing a hat, drinking cold and hot beverages and tying up the hair by questionnaires in front of the National Taiwan Museum of Fine Arts (NTMOFA). Cheng, Lo, and Li [31] surveyed seeking tree shade, cooling under a pavilion, returning to indoor space, quickly moving out of the sun, removing an article of clothing, using an umbrella, wearing a hat and drinking cold and hot beverages, by questionnaires in a campus outdoor environment. Those were unable to adequately allow researchers a comprehensive and in-depth understanding of thermal adaptations.

Thus, further study on thermal adaptation that includes adaptation strategies should be conducted to create an evaluation procedure that can fully measure thermal adaptation in outdoor space. Studies on thermal comfort in urban outdoor environment should include urban plazas [16,23,32], and hence this study collected comprehensive information on the thermal adaptation strategies adopted by urban outdoor plaza users hot and humid Asia. In addition, an evaluation procedure comprising specific categories of outdoor thermal adaptation strategies is proposed to provide a comprehensive reference for future studies on thermal adaptation.

Thermal adaptation surveys are an important research orientation for setting up a comfortable outdoor recreation environment. An effective measurement is a very important research tool for more in-depth and comprehensive understanding of thermal adaptation. This research aims to construct an evaluation procedure of thermal adaptation to outdoor urban plaza conditions in hot and humid Asia. Moreover, thermal adaptation surveys allow researchers to understand people’s response to thermal discomfort, which is crucial to improving thermal comfort in urban outdoor space, especially in hot and humid Asia. However, in past studies, researchers offered few thermal adaptation questionnaire items. In this work will proposes questionnaire items that are appropriate for determining thermal adaptation strategies adopted by these urban plaza users. The evaluation procedure for assessing thermal adaptation strategies and the questionnaire developed in this work can be applied to future research on thermal adaptation strategies adopted by urban plaza users in hot and humid regions of Asia.

## 2. Material and Methods

### 2.1. The Research Location

**Figure 1:**
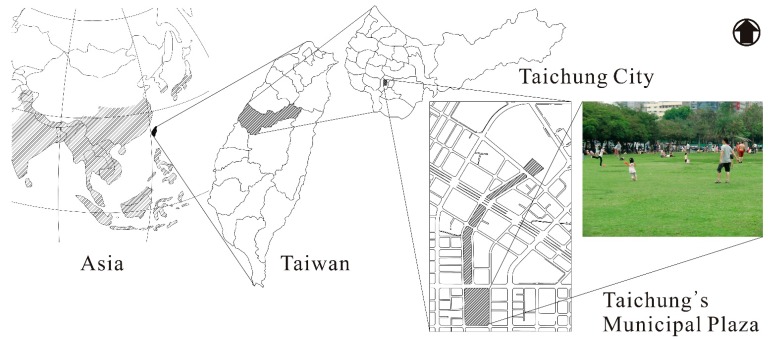
Study site for thermal adaptation strategies adopted by urban plaza users in hot and humid Asia.

This study was conducted in Taichung City (120–40′ E, 24–08′ N, altitude 26 m) in Taiwan’s central region. Situated on the coast of subtropical East Asia, Taichung City is subject to continental and oceanic climate, making the weather in Taichung hot and humid. Statistical data shows that between 1981 and 2010, the average temperature in Taichung City was 23.3 °C [38]. The highest average monthly temperature in July was 33 °C [39], and the number of days with temperature higher than 30 °C was 162.2 [40]. Annual average humidity was 75.6% [41], and the number of rainy days was 113.6 [42]. Hence, the thermal environment of Taichung is characterized by high temperature and high humidity. This research selected Taichung’s Municipal Plaza for the study of adaptation strategies adopted by urban plaza users in humid and hot Asia (Figure 1). The plaza comprises a large grassy area with few shades, and users are more likely to be subjected to environmental comfort level, and hence representative of thermal environment in an outdoor urban plaza. The plaza users were the main subjects of the survey and interviews.

### 2.2. Thermal Adaptation Strategies Adopted by Users in Urban Plaza

Through a literature review, adaptation strategies for stress such as thermal comfort, recreational stress, work and psychological stress, and mental and physical health stress were identified [9,10,15,17,18,34,43,44,45,46,47,48,49,50,51,52,53,54,55,56,57,58,59,60,61,62,63,64,65,66,67,68], and 30 psychological and behavioral thermal adaptation strategies from users interview in study area were compiled (Table 1). Taichung’s Municipal Plaza users were interviewed on their thermal adaptation strategies. On 15–16 October, 2010 when the weather was warmer (SET25.8–29.7 °C), 37 people aged 25–50, including 21 males and 16 females, were interviewed. On 18–19 December 2010 when the weather was cooler (SET 16.2–20.9 °C), 30 people aged 30–40, including 12 males and 18 females, were interviewed. The interview topics were: (1) Does the weather today make you feel uncomfortable? (2) What do you usually do when you feel uncomfortable? Why? (3) How should the environment be modified? 

The interviews were recorded, and the data transformed into text files. Responses to questions such the difference between the weather that day and other days, if the weather felt fine, if the weather felt hot, if the hot weather was perceived as typical, if the plaza was hot as expected, visiting the plaza despite knowledge that the weather would be hot, thermal adaptation methods, personal adaptability, feelings of discomfort, frequency of visit, reasons for visit, duration of visit, activities before the visit, and if duration of visit affects comfort were summarized and tabulated, then compiled and coded responses from interviewees. We coded moving inside (to shade), time of exposure, and perceived control while user’s answer were go to tree shadow, just waiting a moment here, and maintain a happy mood, respectively. Then we coded adjusting clothing, posture, and diet when user’s answered undress, do not exercise, and drink water, respectively. Finally, we coded moving outside and time when user’s answers were go to a supermarket outside the study area, and come back later, respectively. User’s answers fan, wear sunglasses were coded fan, and sunglasses, and so on. In addition, the researchers observed the thermal adaptation strategies adopted by users. Each observation lasted 3–5 minutes to determine the thermal adaptation strategies adopted by the observed plaza user. The observations were recorded using photography, videotaping and field notes. Finally, the interviews produced 21 thermal adaptations strategies for outdoor space (Table 1).

**Table 1:** Thermal adaptation strategies adopted by urban plaza users in hot and humid Asia.

No.	Items	Description	Sources
1	Personal choice	More tolerable because of choice to be exposed to the environment.	Nikolopoulou, 2004 [17]; Nikolopoulou & Steemers, 2003 [18]
2	Expectations	Expects the temperature to change to make it more acceptable.	Cao *et al.*, 2014 [30]; Nikolopoulou, 2004 [17]; Nikolopoulou & Steemers, 2003 [18]
3	Habituation	Diminished feelings due to repeated or long-term exposure to environmental stress.	Brager & de Dear, 1998 [15]; Givoni, 1992 [55]
4	Perceived control	Reduce negative emotional responses by controlling perception of dislike.	Folkman & Lazarus, 1988 [54]; Nikolopoulou & Steemers, 2003 [18]; Field interviews ^1^
5	Past Experience	Recent temperatures help make the environment more acceptable. Such temperature is a given, and a slight increase in temperature is still acceptable.	Humphreys, 1975 [58]; Nikolopoulou & Steemers, 2003 [18]; Field interviews
6	Complaining	Feels more comfortable and bearable after complaining.	Carver *et al.*, 1989 [45]; Djongyang *et al.*, 2010 [47]; Field interviews
7	Naturalness	Viewing it as a natural occurrence makes the wide range of physical environment tolerable.	Nikolopoulou & Steemers, 2003 [18]
8	Time of exposure	Short exposure time makes it tolerable.	Nikolopoulou & Steemers, 2003 [18]; Field interviews
9	Environmental stimulation	People enjoy the environmental incentive, such as the sunshine, wind, and fresh air.	Nikolopoulou & Steemers, 2003 [18]; Field interviews
10	Product transfer	Redefine the experience or change the level of experience, such as: recreating in such weather being a different kind of experience.	Kao, 2007 [34]
11	Rationalization	Convince self that it is not too stressful such as by viewing the weather as a given.	Kao, 2007 [34]; Field interviews
12	Mental disengagement	Not thinking about the discomfort makes it tolerable.	Amirkhan, 1990 [43]; Billings & Moos, 1984 [44]; Carver *et al.*, 1989 [45]; Feifel & Strack, 1989 [51]; Folkman & Lazarus, 1988 [54]; Nowack, 1989 [65]; Field interviews
13	Distraction	Focusing on the activities makes it tolerable.	Billings & Moos, 1984 [44]; Carver *et al.*, 1989 [45]; Dise-Lewis, 1988 [46]; Endler & Parker, 1990 [48]; Folkman & Lazarus, 1980 [52], 1985 [53]; Nowack, 1989 [65]; Patterson & McCubbin, 1987 [67]
14	Endurance ^2^	Just endure.	Dise-Lewis, 1988 [46]; Field interviews
15	Naive optimism	Positive, optimistic thinking makes it acceptable.	Epstein & Meier, 1989 [50]; Folkman & Lazarus, 1985 [53]; Nowack, 1989 [65]; Field interviews
16	Negative thinking	There is nothing one can do about the bad weather or discomfort.	Epstein & Meier, 1989 [50]; Nowack, 1989 [65]
17	Wishful thinking	Thinking that maybe the weather will turn comfortable.	Folkman & Lazarus, 1985 [53]
18	Confrontive *	Face it and endure.	Folkman & Lazarus, 1988 [54]; Field interviews
19	Seeking spiritual support	Tell a friend about it, and get his/her encouragement.	Amirkhan, 1990 [43]; Folkman & Lazarus, 1985 [53], 1988 [54]; Patterson & McCubbin, 1987 [67]
20	Seeking professional support	Ask others about ways to be more comfortable.	Patterson & McCubbin, 1987 [67]
21	Being humorous	Relax and face the weather with humor.	Patterson & McCubbin, 1987 [67]
22	Relaxing	Relax and calm down.	Patterson & McCubbin, 1987 [67]; Field interviews
23	Adjusting clothing(Clothing, hair, hat, umbrella)	Use clothing to adjust to the wide range of weather changes.	Brager & de Dear, 1998 [15]; Djongyang *et al.*, 2010 [47]; Li, 2008 [63]; Lin, T. P., & Lin, Y. T., 2007 [64]; Lin *et al.*, 2013 [33]; Luo *et al.,* 2014 [20]; Nikolopoulou, 2004 [17]; Nikolopoulou & Steemers, 2003 [18]; Tung *et al.*, 2014 [19]; Field interviews
24	Adjustment of activities	Modify personal activities to adapt to the hot environment.	Brager & de Dear, 1998 [15]; Djongyang *et al.*, 2010 [47]; Nikolopoulou, 2004 [17]; Nikolopoulou & Steemers, 2003 [18]
25	Adjust the posture	Modify personal posture to adapt to the hot environment.	Brager & de Dear, 1998 [15]; Djongyang *et al.*, 2010 [47]; Nikolopoulou, 2004 [17]; Nikolopoulou & Steemers, 2003 [18]
26	Adjust your diet(Eating/ drinking hot/ cold food or beverages)	Consume hot or cold food or beverages to adapt to the hot environment.	Brager & de Dear, 1998 [15]; Djongyang *et al.*, 2010 [47]; Li, 2008 [63]; Lin, T. P., & Lin, Y. T., 2007 [64]; Luo *et al.,* 2014 [20]; Nikolopoulou, 2004 [17]; Nikolopoulou & Steemers, 2003 [18]; Field interviews
27	Move inside(To shade)	When the temperature becomes uncomfortable, relocate activity to a more comfortable place.	Kao, 2007 [34]; Lin *et al.*, 2013 [33]; Martinelli *et al.*, 2015 [69]; Tung *et al.*, 2014 [19]; Field interviews
28	Outside displace(Back to the room, quickly through)	Find a place with a more comfortable temperature for the activity.	Kao, 2007 [34]; Field interviews
29	Time displace	If temperature becomes uncomfortable, select another more comfortable time for the activity.	Kao, 2007 [34]
30	Leaving a space	Leave and go to a more comfortable place.	Carver *et al.*, 1989 [45]; Field interviews
31	Fan	Use a hand fan during hot weather.	Lin, T. P., & Lin, Y. T., 2007 [64]; Field interviews
32	Sunglasses	Wear sunglasses if the sun feels too strong.	Field interviews
33	Reduce the time to stay	If temperature becomes uncomfortable at the location, shorten the activity time.	Field interviews
34	Take a deep breath	Take a deep breathe to feel more comfortable.	Field interviews
35	Sunbathe	When the weather is cold, go out into the sunshine.	Field interviews

Notes: **^1^** Field interviews means users were interviewed on their thermal adaptation strategies in Taichung’s Municipal Plaza. **^2^** Assessment by scholars in related fields indicate that these items have a high degree of similarity, and are therefore combined.

The interview and field observation results were then corroborated and added to the thermal adaptation items obtained from the literature review to identify the thermal adaptation strategies adopted by outdoor thermal environment users. To incorporate a broader perspective, all observation and interview data were collected and organized, and a list of 35 thermal adaptation strategies adopted by urban plaza users in hot and humid Asia was subsequently compiled (Table 1). A comprehensive evaluation procedure is needed to determine which of these strategies are applicable to the study of thermal adaptation strategies used in the outdoor thermal environment of hot and humid Asia to help researchers more comprehensively understand thermal adaptation strategies adopted by users.

### 2.3. Thermal Adaptation Evaluation Procedure of Urban Plaza users in Hot and Humid Asia

Questionnaires allow researchers to understand the psychological perception of respondents while measurement tools can effectively quantify theoretical variables whose significance are not easily observable [70]. In particular, questionnaires often explore personal preferences for environments and perception of comfort in a space which not easily to be observed through studying human behavior [16,23,32]. Thus selecting measurable variables for the questionnaires is critical for helping researchers accurately and fully understand the respondents [71]. By compiling questionnaire items from past studies that mainly adopted multivariate methods, appropriate questionnaire items can be constructed [71,72,73,74,75,76,77] using the following procedure: (1) Determine the measurement topic; (2) Establish a questionnaire item bank through literature review; (3) Invite experts to evaluate the preliminary questionnaire item bank; (4) Determine the measurement format, and edit the items; (5) Conduct a pre-sample test; (6) Conduct item, reliability and validity analyses to determine the quality of the questionnaire items; and (7) Use Exploratory Factor Analysis (EFA) and Confirmatory Factor Analysis (CFA) to construct the questionnaire evaluation procedure and determine the appropriate length [70,78]. Through such a procedure, questionnaire items can be effectively formulated, and using this method, this study constructed a thermal adaptation evaluation procedure and formulated appropriate items for determining thermal adaptation strategies.

**Figure 2:**
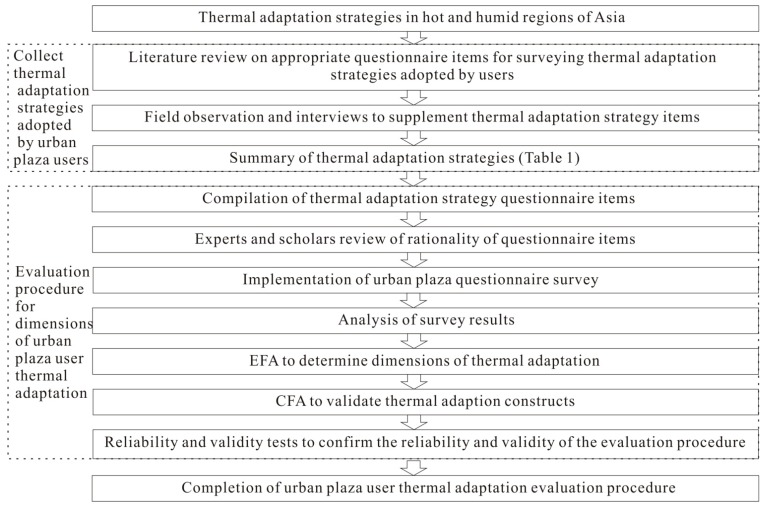
Establishing the thermal adaptation evaluation procedure of urban plaza users in hot and humid Asia.

This study explored the thermal adaptation strategies adopted by urban outdoor plaza users in hot and humid Asia who felt thermal discomfort to determine the dimensions of thermal adaptation. In addition, appropriate thermal adaptation questionnaire items and an evaluation procedure of thermal adaptation by outdoor urban plaza users were developed. Follow the seven steps to set up an evaluation procedure that questionnaire items can be effectively formulated [70,78]. Figure 2 shows the research procedure, where based on literature review, thermal adaptation was first defined and the types of past studies determined. The scope of study in this research was then delineated, namely the thermal adaptation strategies adopted by urban plaza user in hot and humid Asia. The researches then collected information on thermal adaptation strategies adopted by the urban plaza users and reviewed literature on thermal adaptation strategies and stress adaptation in various disciplines to develop appropriate thermal adaptation strategy questionnaire items for urban plaza users. In addition, field observations and interviews were conducted to supplement the inadequacy of literature review, and the obtained thermal adaptation strategies were summarized.

A thermal adaptation construct for urban plaza users was then established, and questionnaire items were formulated based on the collected strategies. After expert and scholar review, the items were compiled into a questionnaire survey. The survey results were tested for item analysis, item difficulty and item discrimination. EFA was applied to determine the dimensions of thermal adaptation, and a preliminary thermal adaption evaluation procedure was constructed. The constructs were tested using CFA, and reliability and validity were also tested, thereby establishing the thermal adaptation evaluation procedure for urban plaza users.

### 2.4. Set up Quantified Questionnaire

Thermal adaptation strategies were collected and compiled into questionnaire items. Using convenient sampling, six experts and scholars with five or more years of landscape research were invited to review the content fit of each strategy item and questionnaire item and the similarity among the strategies, and to help correct ambiguity in the questionnaire items. Issues pointed out by the scholars were corrected, and the reviewed questionnaire items were compiled into a questionnaire survey. During the editing process, the questionnaire items were arranged randomly, and the answer were formatted according to DeVellis [70] and scored on a 5-point Likert Scale to determine the users’ level of agreement or disagreement with adopting certain adaptation strategies. The quantified questionnaire field survey was conducted on 26 March 2011 (15.7 °C average temperature, 73.2% average humidity) and on 30 April, (27.0 °C average temperature, 77.3% average humidity) using convenience sampling. Based on Chiu [78] and DeVellis [70], the expected sample size was 300 persons.

### 2.5. Statistical Analysis

Using the Statistical Package for Social Sciences (SPSS, IBM, Armonk, New York, USA), the sample composition was determined through the descriptive statistics of the respondents’ social economic background. Using item analysis, the questionnaire items were tested for difficulty and discrimination. Appropriateness of the questionnaire item was tested using missing data, item descriptive statistics, comparisons of extreme groups, item-scale (item-total) correlation and factor loading tests, and all items where psychological characteristics were similar or which could not be accurately or effectively tested were eliminated.

Behavioral and psychological adaptation dimensions were analyzed using EFA, and similar attributes were categorized to simplify the complex relationships among the adaptation strategies. Then to simplify the large number of variables [78], missing values in the questionnaire were first substituted with a mean value, followed by principle component analysis for factor extraction. To determine factor correlations while retaining the attributes of the most simplified factors, factor rotation was conducted using the Promax oblique rotation [78]. After factor extraction, Kaiser-Meyer-Olkin (KMO) and Cronbach’s α were used to test the rationality of the factor extraction procedure and factor reliability.

Based on Hsu’s [79] analysis steps, CFA was applied to the constructs obtained from the factor extraction. Structural Equation Modeling (SEM) maximum likelihood (ML) value was used to verify the structural relations among the latent variables, and the accuracy of the relationship between latent variables and observed variables were determined using the LISREL statistical software for CFA [79]. In the analysis, the Modification Index (MI) was used to reconfirm the questionnaires items. Items with MI > 3.84 were eliminated to fulfill the principle of overall fit and item simplicity [79]. For SEM, Hair, Black, Babin, Anderson, and Tatham [80] recommended testing for offending estimates, overall fit criteria, and internal structural fit. Non-offender estimates include positive error variances, standard coefficients ≥0.95, and small standard errors [80]. Using absolute fit measures such as *χ*^2^, df, GFI, SRMR, and RMSEA, incremental fit measures such as NNFI and CFI, and parsimonious fit measures such as PNFI, PGFI and CN were used to verify overall model fit.

In addition, internal structure fit is tested to determine structure reliability and validity. Validity is a test that measures the validity of individual variables in a construct. Bollen [81] believed that validity can be tested by measuring the value and significance of the correlation coefficient between individual latent variables and individual variables. For a variable, its latent variable has one degree of freedom; therefore the absolute *t*-value must be greater than 1.96, and when the *t*-value achieves significance, then the level of validity of that variable is acceptable. Discriminant validity among latent variables can be tested using discriminant validity tests. Using the confidence interval (C.I.) test for paired latent variables, the correlation matrix among the latent variables is tested. The standard deviation for the correlation coefficient of the variables is ±1.96. If confidence interval values do not include 1.00, then latent variables have good discriminant validity [82]. Structural reliability is determined using composite reliability test, where a composite reliability of greater than 0.5 indicates good structural reliability [83].

The questionnaire items on thermal adaptation strategies adopted by urban plaza users in hot and humid Asia were analyzed for difficulty and discrimination using item analysis, categorized using EFA, and tested for item construct using CFA. Reliability and validity tests were also conducted, thereby establishing the thermal adaption construct for urban plaza users in hot and humid Asia.

## 3. Results

### 3.1. Thermal Adaptation Construct for Urban Plaza Users

The 35 thermal adaptation strategies were compiled into questionnaire items which were evaluated by six scholars specializing in landscape. Since “endurance” and “confrontive” items were relatively similar, they were combined. Inappropriate item descriptions were modified, completing the questionnaire construction. Using the resulting 34 thermal adaptation strategies questionnaire items, the questionnaire survey was administered on 26 March, 2011 and 30 April, 2011. A total of 410 questionnaires were distributed, and questionnaires with missing responses, or those with more than 1/3 items left unanswered items were eliminated, resulting in a return of 392 questionnaires, which represents an ineffective rate of 4%. Of the 392 respondents, males accounted for 44.3%, and the largest age group was 21–30 years old (40.8%), followed by 20 or less (20.3%), and 30–40 years old (20.0%).

Results of the questionnaire survey were analyzed for item difficulty and item discrimination using item analysis. Items that received similar responses among all respondents, and items that could not accurately and effectively identify psychological characteristics were deleted, totaling seven items, namely adjusting diet, leaving area, wearing sunglasses, complaining, seeking spiritual support, seeking professional support and reducing the time of stay. The remaining thermal adaptation strategies included 18 psychological adaptation and nine behavioral adaptation items.

Next, EFA was used to determine the constructs for behavioral adaptation and psychological adaptation. Reliability was analyzed using internal consistency method. Items were refined based on three criteria: (1) The corrected item-to-total correlation value is lower than 0.5 [84]; (2) Deleting a particular item can improve the Cronbach’s Alpha of the total correlation [85]; and (3) Whether after factor rotation of the item, the standardized factor loading is lower than 0.3 [86]. Nine behavioral thermal adaptation strategies were retained during the purification, yielding a Cronbach’s Alpha of 0.783, and deleting any of the nine items did not result in any significant increase in overall reliability of the questionnaire. Four psychological thermal adaptation strategies, namely personal choice, expectation, naturalness and being humorous, were deleted. The Cronbach’s Alpha for the remaining 14 items was 0.862, and deleting any of the 14 items did not result in any significant increase in overall reliability of the questionnaire. The standardized factor loadings for the remaining items were greater than 0.3. Therefore, the above purification results indicate that the measurement scale is purified.

Factor extraction analysis of behavioral adaptation items shows a significant correlation coefficient of *p* = 0.000 on the Barlett’s test of sphericity and a KMO value of 0.779 for the construct extraction, indicating that the correlation coefficients between the questionnaire items were sufficient enough for the factors to be used in factor analysis (Appendix Table A1). In addition, based on the eigenvalues >1 and scree test, a total of three factors were extracted. These were labeled displace-oriented behaviors, which explained 36.777% of the total variance; material-aided behaviors, which explained 14.105% of the total variance; and metabolism-related behaviors, which explained 10.831% of the total variance. In addition, each item had a factor loading greater than 0.55, indicating that more than 30% of the variance in the observed items can be explained, and that a high correlation exists between the construct and variables [87].

Factor extraction analysis of the 14 psychological adaptation items shows a significant correlation coefficient of *p* = 0.000 on the Barlett’s test of sphericity and a KMO value of 0.895 for the construct extraction, indicating that the correlation coefficients between the questionnaire items were sufficient enough for the factors to be used in factor analysis (Appendix Table A2). In addition, based on the eigenvalues >1 and scree test, a total of three factors were extracted. These were labeled Intro-control, which explained 37.274% of the total variance; Shift, which explained 8.470% of the total variance; and Passive respondent, which explained 7.507% of the total variance. These three factors explained 53.251% of the total variance, and the factor loading of each item was greater than 0.45, indicating that more than 20% of the variance in the observed items can be explained, and that a high correlation exists between the construct and variables [87]. The above results indicate that the behavioral adaptation strategies and psychological adaptation strategies of urban plaza users have achieved item purification criteria, which proves that the questionnaire was a reliable measuring tool and can be used to determine the thermal adaptation of urban plaza users.

**Figure A1:**
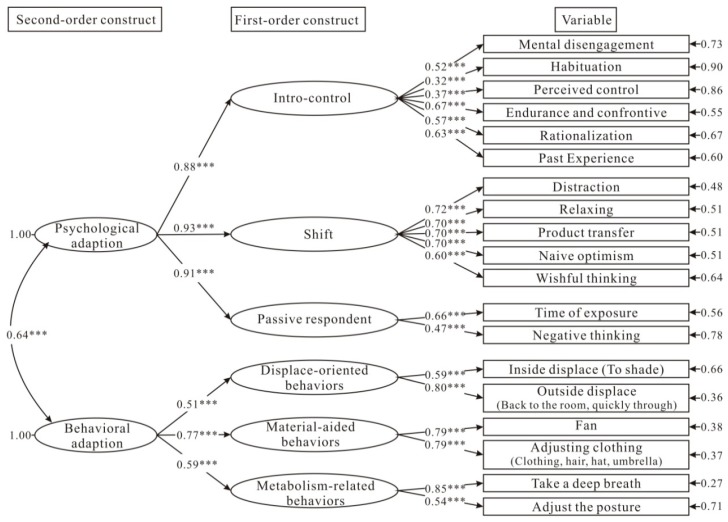
Modified confirmatory factor analysis of urban plaza thermal adaptation.

Based on the above EFA, the thermal adaptation constructs tested using CFA to verify the relationship between the construct and questionnaire item, and the evaluation procedure. Taking into account the modification index, non-offending estimates and goodness of fit, four items, namely environmental stimulation, adjustment of activities, time displace, and sunbathe were deleted to achieve overall goodness of fit and item simplification. After purification, the psychological adaptation and behavioral adaptation evaluation procedure constitute the second-order construct, which encompassed three first order constructs comprising a total of 19 variables (Appendix Figure A1). A goodness of fit was achieved after modification, except that *χ*^2^ failed to meet the criteria for best fit (Appendix Table A3). However, Hatcher [88] pointed out that a large sample causes an overly high *χ*^2^, which is therefore acceptable in the overall model. Results of the standardized factor loading (Appendix Figure A1) show that in the behavioral adaption dimension, material-aided behaviors (β = 0.77) was the most representative construct; in the psychological dimension, Shift (β = 0.93) showed the most optimal result.

Reliability and validity analyses showed that standardized factor loading for all the questionnaire items were significant (*t*-value > 3.291) (Appendix Figure A1), indicating that each item of measurement achieved acceptable validity. Analysis for discriminant validity (Appendix Table A4) shows that the confidence intervals of the correlation matrix did not include 1, indicating that intro-control, shift, passive respondent, and displace-oriented, material-aided and metabolism-related behavioral constructs showed excellent discriminant validity. Reliability analysis show that composite reliability was higher than 0.5 (0.5–0.9) for each construct, indicating that the evaluation procedure has acceptable reliability.

### 3.2. Verifying the Thermal Adaptation Strategies of Urban Plaza Users Hot and Humid Asia

**Figure 3:**
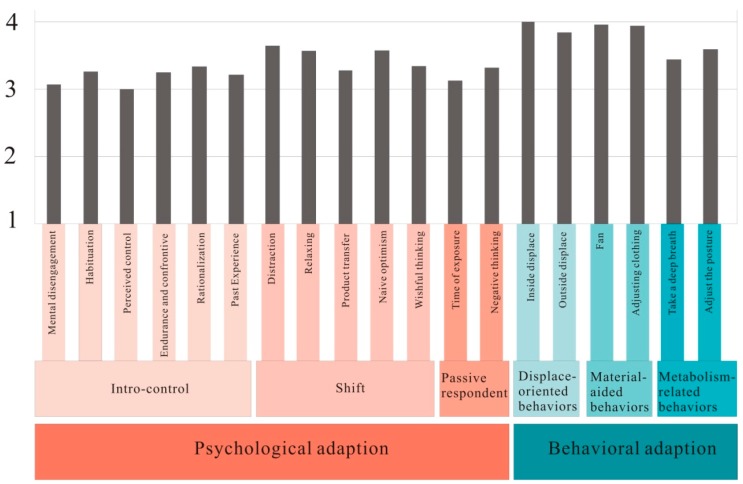
Distribution frequency of thermal adaption strategies adopted by users of Taichung’s Municipal Plaza.

Using Taichung’s Municipal Plaza for the empirical case study, the thermal adaptation strategy survey was administered, and a distribution frequency of adopted strategies was constructed (Figure 3) to show the thermal adaptations of urban plaza users in hot and humid Asia. Analysis results show that the park users in hot and humid Asia most frequently adopted behavioral adaptation strategies to increase their thermal comfort. Among these strategies, displace-oriented behaviors and material-aided behaviors were more frequently used. In the displace-oriented behaviors, inside-displace was most frequently used. Users resorted to psychological adaptation strategies only when behavioral strategies could not be adopted. Among the psychological thermal adaptation strategies, shift was most frequently used, among which distracting oneself with the ongoing activity was frequently used to help endure the thermal discomfort.

## 4. Discussion

This study aimed to determine the thermal adaptation strategies adopted by urban plaza users in hot and humid Asia when they feel thermal discomfort. The obtained strategies were then used to establish a construct for thermal adaption in outdoor urban plazas in hot and humid Asia. Questionnaire items appropriate for surveying thermal adaptation strategies adopted by outdoor urban plaza users in hot and humid Asia were proposed. Using Taichung’s Municipal Plaza for the empirical study, these strategy items were verified. Results of the study are discussed in the following sections.

### 4.1. The Thermal Adaptation Strategies Adopted by Users When They Felt Thermal Discomfort

Many past empirical studies focused on behavioral and psychological thermal adaptation strategies [16,18,19,32,33,69,89], indicating the realization that the thermal adaptation strategies of users are important issues. These strategies influence the perception of thermal comfort of urban plaza users and their willingness to participate in urban plaza activities, and hence the importance of thermal adaptation strategy surveys. What thermal adaptation strategies do urban plaza users adopt when they feel thermal discomfort? Literature review indicates that the most frequently studied thermal adaptation items were behavioral strategies such as adjusting clothing (clothing, hair hat, umbrella), adjusting activities, adjusting posture, adjusting diet (eat and drink hot or cold food or beverages), moving inside (going under a tree or seeking shade), moving outside (passing through quickly), time displace, and leaving [15,17,18,19,20,33,34,45,47,63,64].

The urban plaza users surveyed in this study were also more willing to adopt most of these thermal adaptation strategies (Figure 3). However not all of these items were applied in this research, and a few items were selected for further study. Behavioral strategies delineated from literature review, such as adjusting clothing, adjusting diet, moving inside, moving outside, leaving and using a hand fan were verified through field observation and interviews in this study. In addition, other behavioral strategies not found in previous studies were mentioned by the urban plaza users in this study, such as wearing sunglasses, reducing time of stay, taking a deep breath, and sunbathing. Among these thermal adaptation strategies, using a hand fan was a strategy most frequently adopted by the urban plaza users. Taking a deep breath did not rank high among the behavioral strategies of users, but was adopted more frequently than most psychological adaptation strategies.

Psychological adaptation strategies were often mentioned in previous studies, and literature review reveals a variety of psychological adaptation questionnaire items [15,17,18,34,37,43,44,45,46,47,48,49,50,51,52,53,54,55,58,65,67]. Applying these items to the field observation and interviews in this study showed that the urban plaza users also mentioned many of these items, such as perceived control, past experience, complaining, time of exposure, environmental stimulation, rationalization, mental disengagement, endurance, positive thinking, confrontation, and relaxation. Analysis of results showed that although psychological adaptation strategies were mentioned more frequently than behavioral items in literature review, case studies and interviews (Table 1), they were less likely to be adapted than behavioral strategies. This finding is consistent with Wu’s [37] viewpoint. Behavioral and psychological thermal adaptation strategies were different [15,18]. Behavioral thermal adaption is a form that has an instantaneous effect following an action, while psychological adaptation is a gradual change in perception of the environment.

Thermal adaptation strategies for building complexes showed that behavioral and psychological adaptations were equally valued [15]. The discrepancy could be due to the greater likelihood of using behavioral adaptation and less require of thermal comfort in outdoor thermal environments [15,18,30,90]; therefore, the difference compared to the thermal adaptation strategies usually adopted for indoor environment. Moreover, geographic locations do matter different climate conditions for areas of people’s origins often impact their decisions and perception on thermal adaptation strategies. For example, people live in Taiwan better able to tolerate hot weather but worst able to tolerate cold weather than live in American [91].

This study also found that the thermal adaptation strategies adopted by urban plaza users in hot and humid Asia included 35 items, of which 13 items were behavioral adaptations and 22 items were psychological adaptations. As shown in Appendix Table A1 and Table A2, users were more likely to adopt behavioral adaptation strategies than psychological adaptations strategies. The mean value showed that when urban plaza users in hot and humid Asia felt thermal discomfort, they mainly responded with behavioral adaptation strategies, and only resorted to psychological adaptations strategies upon failure to completely alleviate thermal discomfort.

### 4.2. Thermal Adaptation Strategies Most Frequently Adopted by Urban Plaza Users in Hot and Humid Asia

Conducted in Taichung’s Municipal Plaza, this empirical study found that the thermal adaptation strategies most frequently adopted by urban plaza users in hot and humid Asia were move-oriented behaviors and material-aided behaviors (Figure 3). In terms of mean likelihood for using move-oriented behaviors, moving inside had a mean of M = 4.02; and moving outside, M = 3.85. For material-aided behaviors, the mean value for using hand fan was M = 3.96; and adjusting outfit, M = 3.94. Evidently, when urban plaza users felt thermal discomfort, they first chose to move to an environment that afforded greater thermal comfort or adjust their outfits such as clothes, hats, umbrellas and fans to improve their thermal comfort. The users adopted psychological adaptations such as focusing on the activities to help them endure the thermal discomfort of the environment only when they are unable to adopt behavioral adaptation.

Among the many thermal adaptation strategies, leaving a place can most directly solve the problem of thermal discomfort, and hence displace-oriented behaviors were most frequently adopted by users, among which moving inside was more frequently used than moving outside. Since users at the Municipal Plaza had chosen to be at the plaza for the activities, they were less likely to leave, and for those who stayed, their choice of displacement strategies included moving inside such as going under a tree or a shady area for their thermal adaptation. This finding is consistent with Lin *et al.* [33], Martinelli *et al.* [69], Tung *et al.* [19] viewpoint. Thus, to improve the thermal comfort of plaza users, providing more shaded areas or trees can enhance move-oriented behaviors [19,92,93].

The Municipal Plaza users also showed a high likelihood of adopting material-aided behaviors, which ranked only lower than moving inside strategies. This is because if the users were engaged in an activity, they could not move away to a shady area, and would instead add or remove an article of clothing, tie up their hair, wear hats, hold umbrellas, or fan themselves for thermal comfort [31,32,64]. These thermal adaptation strategies are quicker and more effective for improving thermal discomfort than metabolism-related behavioral adaptation and psychological adaptation. Therefore, to improve the thermal comfort of plaza users, vendors for thermal merchandise should be available in or around the plaza to facilitate material-aided adaptation for plaza users.

Among metabolism-related behaviors, taking a deep breath showed a mean likelihood of M = 3.44, and adjusting posture a mean of M = 3.59. Although these strategies scored lower than other behavioral strategies, they scored higher than most psychological adaptation strategies (Figure 3). Of these two behaviors, adjusting posture scored higher than taking a deep breath because in a cold thermal environment, passive activities could be changed to more dynamic activities such as running while in a hot thermal environment, more dynamic activities could be changed to more passive activities such as meditation. Since this particular thermal adaptation strategy is more widely applicable, it was more likely to be adopted by users. Nevertheless, taking a deep breath can greatly alleviate thermal discomfort. Previous studies showed that breathing can affect thoughts, and deep breathing can relieve stress [94,95], enhance oxygen intake and thereby increase metabolism, reduce sympathetic nervous division and thereby reduce heart rate and blood pressure, resulting in a more restful physical state [96]. Thus, taking a deep breath facilitates a more restful state, and helps relieve thermal discomfort when the thermal environment is hot. Therefore, to help the Municipal Plaza users adopt metabolism-related behavioral adaptations to alleviate their thermal discomfort, more rest platforms, comprehensive sports facilities or trails could be constructed to provide plaza users with facilities practicing metabolism-related behavioral adaptations.

The Taichung’s Municipal Plaza study found that users were less willing to adopt psychological adaptation strategies than behavioral adaptations. However, when behavioral adaptation was not feasible, users rely on controlling thoughts to enhance thermal comfort without having to implement any behavioral actions. The most frequently adopted psychological adaptation strategy was shift, which mainly included distraction, relaxing and naïve optimism to alter personal mood and render the thermal environment more tolerable. Billings and Moos [44], Carver *et al.* [45], Dise-Lewis [46], Endler and Parker [48,49], Epstein and Meier [50], Folkman and Lazarus [52,53], Kao [34], Nowack [65], Patterson and McCubbin [67], and Wu [37] also found that these methods are effective for diverting stress.

However, among the psychological adaptation strategies, intro-control and passive respondent were less likely to be adopted. Unlike shift strategies which enhanced thermal adaptation, intro-control strategies such as rationalization and habituation, and passive-respondent strategies such as feeling that nothing could be changed or thinking that exposure time is limited anyway merely helped users endure thermal discomfort, and are therefore less likely to be adopted by users. Therefore, to help the Municipal Plaza users adopt metabolism-related behavioral adaptations to alleviate their thermal discomfort, scenic facilities such as cultivating more plants that flower and attract birds and butterflies, waterscape with flowing water, landscape lighting on trees or pavilions could be constructed in the Plaza to help users to shift their mood [97]. Poems and slogans reminding the Municipal Plaza users of psychological adaptation strategies, or media conducive for shifting mood could also be installed to help users with psychological adaptation.

### 4.3. Establishing the Thermal Adaptation Strategies Construct

Thermal adaptation strategies adopted by urban plaza users in hot and humid Asia included six constructs. The behavioral adaptation dimension included three behavioral adaptation constructs, namely moving-oriented behaviors, material-aided behaviors and metabolism-related behaviors. The psychological adaptation dimension included three psychological adaptation constructs, namely intro-control, shift, and passive-respondent.

Move-oriented behavioral construct, defined as users moving to a more comfortable place to continue with an ongoing activity to increase thermal comfort when feeling thermal discomfort, comprised two thermal adaptation strategies, namely moving inside and moving outside. Material-aided behavioral construct, defined as users adding or removing articles of clothing (such as adjusting clothes, tying up hair, wearing a hat, or holding an umbrella) to alleviate thermal discomfort, comprised two thermal adaptation strategies, namely using a fan and adjusting clothing. Metabolism-related behavioral construct, defined as modifying physical metabolism (such as taking a deep breath and altering posture) to alleviate thermal discomfort, comprised two thermal adaptation strategies, namely taking a deep breath and altering posture.

Intro-control construct, defined as using self-control to enable acceptance of an uncomfortable thermal environment (such as not thinking about the discomfort, and just going through with it), comprised six thermal adaptation strategies, namely mental disengagement, habituation, perceived control, endurance and confrontive, rationalization, and past experience. Shift construct, defined as altering one’s mood to accept an uncomfortable thermal environment (such as positive and optimistic thinking, and paying attention to the ongoing activities), comprised five thermal adaptation strategies, namely distraction, relaxation, product transfer, naïve optimism, and wishful thinking. Passive respondent construct, defined as passively accepting an unchangeable uncomfortable thermal environment, comprised two thermal adaptation strategies, namely time of exposure and negative thinking. Using these six adaptation strategies constructs to explain the psychological and behavioral thermal adaptation strategies adopted by urban plaza users in hot and humid Asia yielded an excellent total variance (50% or higher), thereby indicating that these constructs have satisfactory construct reliability and construct validity.

The thermal adaptation constructs delineated by this study are a reasonable summary of the thermal adaptation strategies adopted by urban plaza users. These constructs not only verified the behavioral and psychological thermal adaptation constructs found in previous studies [15,34,37,47,62], but further confirmed the concept of multi-dimensionality in behavioral and psychological adaptations. This study found that among the urban plaza thermal adaptation strategies, the behavioral adaptation dimension included three constructs comprising six variables, while the psychological adaptation dimension included three constructs comprising 13 variables. The behavioral thermal adaptation strategies found in this study are consistent with the results of studies on indoor and outdoor behavioral adaptations by Brager and de Dear [15], Cheng *et al.* [31], Lin [32], Lin *et al.* [33], Luo *et al.* [20], and Tung *et al.* [19], rendering these results suitable for exploring the thermal environment of urban plazas in hot and humid Asia (Appendix Figure A1). Similarly, in the psychological adaptation dimension, the constructs are consistent with the results of studies by Brager and de Dear [15], James and Norman [59], Kao [34], Nikolopoulou [17], and Nikolopoulou and Steemers [18], as shown in Appendix Figure A1, where psychological adaptation variables are re-linked to the thermal environment of urban plazas. These urban plaza thermal environment constructs are appropriate for determining a thermal adaptation evaluation procedure, which is an important contribution of this study toward clarifying and more comprehensively linking urban plaza thermal adaptation concepts.

### 4.4. Constructing the Thermal Adaptation Strategies Questionnaire Items

Based on the thermal adaptation evaluation procedure, this study proposed a simplified version (Table 2) and a detailed version (Table 3) of questionnaires appropriate for studying the thermal adaptation of urban plaza users hot and humid Asia.

Six major constructs were adopted to formulate the simplified 6-item version (Table 2), where behavioral and psychological adaptation tests contain three items each. The behavioral adaptation comprise three major constructs, namely move-oriented behaviors, material-aided behaviors and metabolism-related behaviors; the psychological adaptation comprise three major constructs, namely intro-control, shift and passive respondent. The questionnaire covers a comprehensive range of thermal adaptations, but is short, and therefore less burdensome to respondents [70]. It is easy to fill, and therefore offers greater flexibility for future use. It is also appropriate for research involving thermal adaptation, such as studies by Cheng *et al.* [31], Kao [34], Lin [32], Lin *et al.* [16], Lin *et al.* [33], and Nakano & Tanabe [23], which contained few questionnaire items on behavioral adaptation. Hence, the results of this study can help such studies obtain more comprehensive information.

**Table 2:** Thermal adaption questionnaire items for urban plaza users in hot and humid Asia (simplified version).

Thermal Adaptation Construct	Thermal Adaptation Questionnaire Item
Move-oriented behavior	I will find a more comfortable place for the activity.
Material-aided behavior	I will add or remove articles of clothing to relief thermal discomfort (such as clothes, hat, umbrella or fan).
Metabolism-related behavior	I use change my body’s metabolism to relief thermal discomfort (such as taking a deep breathe or changing posture).
Intro-control	I will use self-control to help myself accept the thermal discomfort (such as not thinking about the discomfort or enduring the discomfort).
Shift	I will change my mood to help myself accept the thermal discomfort (such as positive thinking or focusing on the ongoing activity).
Passive respondent	I think the thermal discomfort cannot be helped, and therefore, I can only passively accept it.

In addition, based on the thermal adaptation evaluation procedure, 13 psychological adaption and six behavioral adaptation items were proposed for constructing the detailed version questionnaire (Table 3). The questionnaire items on urban plaza in hot and humid Asia are comprehensive, and verified by CFA, reliability and validity tests. The evaluation procedure showed excellent construct reliability and validity, and the evaluation procedure and item content are comprehensive and reasonable (Appendix Table A1, Table A2, Table A3 and Table A4, Appendix Figure A1). However, the detailed version contains 19 items, and therefore requires more respondent time and energy, resulting in less willingness to participate in the survey. Moreover, respondents may tire of answering the questions, resulting in errors. However, this longer version has a more stable α value, and therefore more reliable [70]. This measurement is appropriate for studies that primarily focus on thermal adaptation. Previous studies have shown that thermal adaptation survey is an important future research orientation, and this measurement is a very important research tool for more in-depth and comprehensive understanding of thermal adaptation. Based on the subjects and research needs, future studies can select the simplified or detailed version for questionnaire survey.

**Table 3:** Thermal adaption questionnaire items for urban plaza users in hot and humid Asia (detailed version).

Thermal Adaptation Variables	Thermal Adaptation Questionnaire Item
	I will stay in a more comfortable place (such as a cooler or warmer place).
Move outside	I will go to a more comfortable environment for recreation.
Fan	If the day is hot, I will use a hand fan.
Adjusting clothing	I will add or remove a clothing item, or tie up my hair, wear a hat, or use an umbrella.
Take a deep breath	I will take a deep breath to help myself feel more comfortable.
Adjust the posture	I will change my posture to help myself feel more comfortable.
Mental disengagement	Not thinking about the discomfort will make it tolerable.
Habituation	Getting used to it will relief the discomfort.
Perceived control	I will tell myself that the weather does not feel uncomfortable.
Endurance and confrontive	I will face it and get over it.
Rationalization	Such weather is normal here, and it’s not particularly bad.
Past Experience	Compared to the past, today’s weather is actually quite good and acceptable.
Distraction	Focusing on the ongoing activity helps make it tolerable.
Relaxing	I will relax and calm down.
Product transfer	The uncomfortable weather is another kind of experience.
Kind of Naive optimism	Thinking positively and optimistically makes it acceptable.
Wishful thinking	I think maybe the weather will become more comfortable in a while.
Time of exposure	I am not staying long, so the discomfort is acceptable.
Negative thinking	The bad weather and discomfort cannot be helped.

## 5. Conclusions

Thermal adaptation surveys allow researchers to understand people’s response to thermal discomfort, which is crucial to improving thermal comfort in urban outdoor space, especially in hot and humid Asia. However, in past studies, researchers offered few thermal adaptation questionnaire items. In this study, 35 items of thermal adaptation strategies adopted by urban plaza users were derived from literature review and field observations and interviews with 67 participants. These items were refined using item analysis, EFA, CFA, and reliability and validity tests. Thirteen psychological adaptation and six behavioral adaptation items were delineated to construct a questionnaire, which was tested in Taichung’s Municipal Plaza. Results indicated that behavioral adaptation strategies were more frequently used to enhance thermal comfort, among which moving-oriented behaviors and material-aided behaviors were most frequently adopted. When behavioral adaptation could not be adopted, psychological adaptations were used, the most frequent being shift strategies, which included distraction or focusing attention on the ongoing activity to help tolerate thermal discomfort. Last, a simplified version and detailed version of the thermal adaptation evaluation procedure appropriate for urban plaza users in hot and humid Asia were proposed.

This evaluation procedure should be applied to analyze user’s behavior at open space before landscape engineering. Information from the behavior analysis will be useful for landscape planner to design a comfort outdoor open space in urban areas. Moreover, the procedure could be applied to the post-occupancy evaluation while outdoor open space have been constructed for a few years. Results of the post-occupancy evaluation could offer practical insights to government for how to remodel outdoor open space. One of the limitation of thermal adaptation evaluation procedure is that the sampling locations of this study is only in Taiwan. A cross-country comparing research should be done to approve its reliability and validity before applied to other Asia’s Hot-Humid Regions.

## Appendix

**Table A1:** Results of exploratory factor analysis of behavioral adaptation to the thermal environment of urban plaza.

Behavioral Adaptation Strategies to the Thermal Environment of Urban Plaza	Mean	Behavioral Adaption		
		Displace-Oriented Behavior	Material-Aided Behavior	Metabolism-Related Behavior
Move inside (to shade)	4.02	0.784	0.345	0.286
Move outside (back to the room, quickly move through area)	3.85	0.761	0.302	0.143
Adjustment of activities	3.93	0.682	0.443	0.414
Time displacement	3.61	0.678	0.349	0.213
Fan	3.96	0.311	0.820	0.135
Adjusting clothing (clothing, hair, hat, umbrella)	3.94	0.405	0.809	0.332
Sunbathe	4.01	0.411	0.623	0.335
Take a deep breath	3.44	0.232	0.257	0.893
Adjust the posture	3.59	0.338	0.297	0.889
Eigenvalue		3.310	1.269	0.975
Explained variance		36.777	14.105	10.831
Cumulative explained variance		36.777	50.883	61.714
Factors mean		3.85	3.97	3.52
Kaiser-Meyer-Olkin = 0.779 Cronbach’s Alpha = 0.783				

Notes: 1. Sample sizes is 2076 and observations is 392 persons; 2. Thermal adaptation items were scored on a 5-point Likert Scale (“1” = strongly disagree, “5” = strongly agree); 3. Through item analysis, 4 items that received similar responses among all respondents, namely adjusting diet, leaving, wearing sunglasses and reducing staying time, and items that could not accurately and effectively identify psychological characteristics were deleted.

**Table A2:** Results of exploratory factor analysis of psychological adaptation to the thermal environment of urban plaza.

Psychological Adaptation Strategies to the Thermal Environment of Urban Plaza	Mean	Psychological Adaption		
		Intro-Control	Shift	Passive Respondent
Mental disengagement	3.05	0.738	0.496	0.074
Environmental stimulation	2.99	0.725	0.512	0.226
Habituation	3.26	0.719	0.325	0.367
Perceived control	2.99	0.703	0.391	0.102
Endurance and confrontive	3.24	0.652	0.463	0.412
Rationalization	3.34	0.633	0.511	0.415
Past Experience	3.21	0.600	0.401	0.338
Distraction	3.65	0.417	0.821	0.243
Relaxing	3.57	0.445	0.811	0.230
Product transfer	3.28	0.565	0.757	0.193
Naive optimism	3.57	0.534	0.745	0.373
Wishful thinking	3.35	0.358	0.475	0.105
Time of exposure	3.12	0.247	0.280	0.776
Negative thinking	3.32	0.255	0.166	0.754
Eigenvalue		5.218	1.186	1.051
Explained variance		37.274	8.470	7.507
Cumulative explained variance		37.274	45.744	53.251
Factors mean		3.15	3.48	3.22
Kaiser-Meyer-Olkin = 0.895 Cronbach’s Alpha = 0.862				

Note 1: Sample sizes are 2076 and observations are 392 persons; Note 2: Thermal adaptation items were scored on a 5-point Likert Scale (“1” = strongly disagree, “5” = strongly agree); Note 3: Through item analysis, 3 items that received similar responses among all respondents, namely complaining, seeking spiritual support and seeking professional support, and items that could not accurately and effectively identify psychological characteristics were deleted. Then through EFA, 4 items, namely personal choice, expectation, naturalness and being humorous were deleted because their total correlation value or standardized factor loading were too low, or because their deletion increased the total Cronbach’s Alpha; Note 4: Assessment by scholars in related fields indicated that the “enduring” and “confrontive” items have a high degree of similarity, and are therefore combined.

**Table A3:** Modified thermal adaptation goodness of fit.

Fit Index	*χ*^2^	*χ*^2^/df	GFI	SRMR	RMSEA	NNFI	CFI	PNFI	PGFI	CN
Criteria	*p* > 0.1	<3	>0.9	<0.08	<0.08	>0.9	>0.9	>0.5	>0.5	>200
Adaptation Factor	363.9, *p* < 0.1	2.51	0.91	0.06	0.06	0.94	0.95	0.78	0.69	202

Notes: 1. Sample sizes are 2076 and observations are 392 persons; 2. a. *t*-value > 1.96 (*p* < 0.05, denoted by *); *t*-value > 2.576 (*p* < 0.01, denoted by **); *t*-value > 3.291 (*p* < 0.001, denoted by ***).

**Table A4:** Discriminant validity of thermal adaptation strategies.

Latent Variable	Intro-Control	Shift	Passive Respondent	Displace-Oriented Behavior	Material-Aided Behavior		Metabolism-Related Behavior
Intro-control	1						
Shift	0.81 ^a^ [0.73,0.89] ^b^	1					
Passive respondent	0.85 [0.71,0.99]	0.81 [0.69, 0.93]	1				
Displace-oriented behavior	0.13 [−0.01,0.27]	0.25 [0.11, 0.39]	0.07 [-0.09, 0.23]	1			
Material-aided behavior	0.42 [0.30,0.54]	0.57 [0.47, 0.67]	0.47 [0.33, 0.61]	0.36 [0.22, 0.50]	1		
Metabolism-related behavior	0.31 [0.17, 0.45]	0.34 [0.22, 0.46]	0.32 [0.16, 0.48]	0.55 [0.41, 0.69]	0.40 [0.28, 0.52]	1	

Notes: **^1^** Sample sizes are 2076 and observations are 392 persons; **^2^** a: correlation coefficient; b: confidence intervals.
